# Cornea organoids from human induced pluripotent stem cells

**DOI:** 10.1038/srep41286

**Published:** 2017-01-27

**Authors:** James W. Foster, Karl Wahlin, Sheila M. Adams, David E. Birk, Donald J. Zack, Shukti Chakravarti

**Affiliations:** 1 Department of Medicine, Johns Hopkins School of Medicine, Baltimore, MD, USA; 2 Department of Ophthalmology, University of California San Diego, La Jolla, CA, USA; 3 Department of Molecular Pharmacology & Physiology, University of South Florida, Tampa, FL, USA; 4 Department of Ophthalmology, Johns Hopkins School of Medicine, Baltimore, MD, USA; 5 Department of Cell Biology, Johns Hopkins School of Medicine, Baltimore, MD, USA.

## Abstract

The cornea is the transparent outermost surface of the eye, consisting of a stratified epithelium, a collagenous stroma and an innermost single-cell layered endothelium and providing 2/3 of the refractive power of the eye. Multiple diseases of the cornea arise from genetic defects where the ultimate phenotype can be influenced by cross talk between the cell types and the extracellular matrix. Cell culture modeling of diseases can benefit from cornea organoids that include multiple corneal cell types and extracellular matrices. Here we present human iPS cell-derived organoids through sequential rounds of differentiation programs. These organoids share features of the developing cornea, harboring three distinct cell types with expression of key epithelial, stromal and endothelial cell markers. Cornea organoid cultures provide a powerful 3D model system for investigating corneal developmental processes and their disruptions in diseased conditions.

The cornea is the outer protective layer of the eye that provides 2/3 of the refractive power required to focus light on the photosensitive retina^1^. The bulk of the cornea is a collagenous extracellular matrix (ECM) layer with embedded keratocytes, cells that produce and maintain this ECM, bound by an outer layer of stratified epithelium and an innermost layer of endothelium. The development of the cornea begins with the surface ectoderm that overlies the lens vesicle. Inductive interactions between the lens vesicle and the ectoderm drive migration of neural-crest derived mesenchymal cells that will form the endothelium and finally the stromal keratocytes, into the space between the lens vesicle and the developing corneal epithelium^2^,^3^,^4^,^5^,^6^.

Despite its relative simplicity, *in vitro* modeling of the cornea has traditionally used individual constituent cell types of the epithelium, stroma or the endothelium^7^,^8^. In the context of disease modeling such as in Fuch’s dystrophy^9^ or Keratoconus^10^ (complex diseases with poorly understood etiologies), this approach does not consider the influences of other cell types in investigating disease features *in vitro*. In addition, studies of the ECM types they produce usually require long-duration cultures to elicit potential differences underlying disease. Therefore, we sought to develop three-dimensional (3D) corneal organoids comprising all the component cell types of the cornea in a stable system. This provides a unique opportunity to investigate cellular disease phenotypes for long periods of time, including developmental aspects, and incorporates influences of interactions between different cell types and the ECM.

Recent efforts have made tremendous gains in developing self-organizing 3-dimensional organoids from embryonic stem cells, and subsequently later from induced pluripotent stem cells (iPSC)^11^,^12^,^13^,^14^,^15^,^16^. These studies will impact human disease modeling, drug testing and possibly organ replacement treatments^17^. Signals that regulate early development of the eye field, optic vesicle to the optic cup have been used to develop retinal organoids from embryonic stem cells *in vitro*^12^,^18^. More recently, similar regulatory signals have been used to develop human retinal organoids from iPSCs to produce optic vesicle and optic cup-like structures with a neural retina bearing a cellular composition similar to the mature human retina^19^. Here we describe derivation of cornea organoids from human fetal fibroblast-derived iPSCs as they were being used to develop 3D retina-like structures (Wahlin, under review). Wahlin and coworkers developed a modified approach to generate 3D retinas containing cells that resemble photoreceptors, including rods and cones with outer-segment like structures. Briefly, iPSCs were used to promote an anterior neural commitment using a matrigel ECM, Wnt signaling inhibition, and manual dissection of the developing neural vesicles followed by exposure to retinoic acid and brief Notch signaling inhibition. While this approach reliably produced 3D optic vesicles and other anterior neural vesicle, in a small (but reproducible) number of cases the aggregated cells developed into translucent organoids. Results presented here show that these translucent organoids reflect corneal features. Most importantly, we show that these organoids develop into laminar organized structures resembling corneal epithelium, stroma and endothelium and accumulate stromal ECM collagen fibrils.

## Results

### Cornea organoid morphology

**Figure 1:**
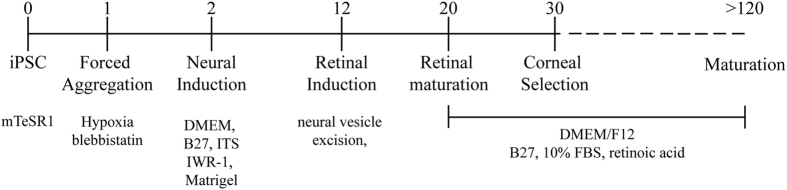
Outline of corneal organoid derivation method. IMR90.4 HPS cells were induced to differentiate to optic vesicles; these were subsequently isolated and underwent further differentiation. By day 30 corneal organoids were identifiable and further differentiated for over 120 days.

**Figure 2:**
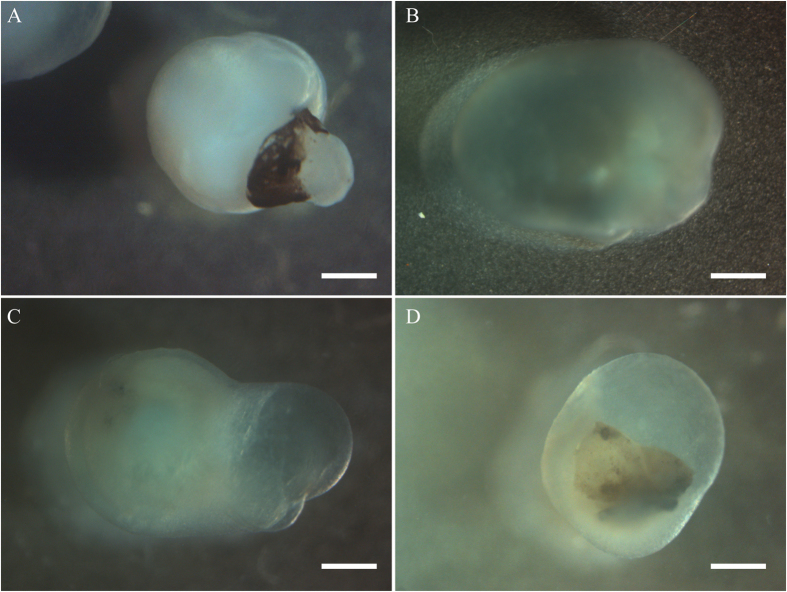
Gross morphology of organoids. Bright field observations of four organoids (**A**–**D**). After 3 months in culture these appeared translucent with a dense (**A**) or a clear (**B**–**D**) center, sometimes with a pigmented end (**C**). Scale bar 200 μm.

Methodological details of producing cornea organoids (C-ORG) from iPSCs are outlined in Fig. 1. The morphology of 4 different C-ORGs, harvested on day 120 is shown in Fig. 2. The C-ORGs ranged in size from 0.5 mm to 1.5 mm, spherical to oblong in shape and were translucent to almost transparent (Fig. 2A–C) or somewhat dense centrally (Fig. 2D). The translucent/transparent C-ORGs appeared to be fluid-filled. The C-ORGs do not show the structural complexity and the cellular organization of retinal optic cups in bright field images or in immunofluorescence staining of sections as presented later. The C-ORGs lack gross anterior to posterior orientation as seen in the whole eye, where the cornea forms one end of the axis and the retina/optic cup the other end. Some C-ORGs have a small stock at one end pigmented by the presence of presumptive retinal pigment epithelium (RPE) cells (Fig. 2A and C).

### Corneal gene expression

**Figure 3:**
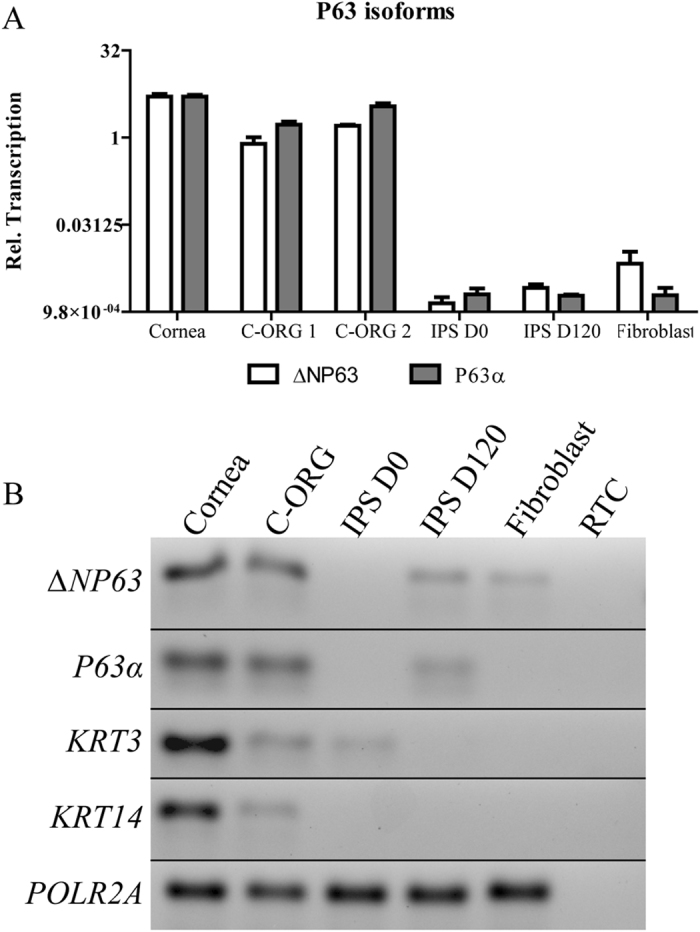
Expression of *P63* isoforms and epithelial markers. Human cornea (Cornea), organoid cultures (C-ORG 1 and 2), and human corneal fibroblasts, were analyzed by qRTPCR (**A**). Both ΔNP63 and p63α isoforms were expressed in the cornea and the two individual organoids, C-ORG1 and 2. The qPCR products for the p63 isoforms and additional KRT3 and 14 are shown (**B**). *POLR2A* was used as a housekeeping gene.

We investigated expression of key corneal genes that mark epithelial cells and epithelial stratification, and stromal keratocytes. For this purpose we used quantitative reverse transcription PCR (RT-PCR) on total RNA extracted from individual (not pooled) C-ORGs, donor corneas, as well as cultured corneal fibroblasts as references (Fig. 3 and Supplemental Figure 1). The trans-activating TA p63 isoforms are transcribed from an alternative upstream promoter and expressed during early embryogenesis^20^, while the ΔNp63 isoforms, lacking two exons from the 5′ end are expressed in the differentiating epithelia^21^,^22^,^23^. We detected transcripts for ΔNP63 and p63α in the adult cornea and in the C-ORG total RNA extracts but not in the iPSC or stromal fibroblasts (Fig. 3A); although a faint p63α band is detectable in the pCR products run in a gel (Fig. 3B). We also investigated expression of corneal epithelial keratins (*KRT*)’ *KRT*3 and *KRT*14 and these appear as bands of expected sizes in C-ORG samples (Fig. 3B). KRT 12 is known to be present in the suprabasal differentiated epithelial cells^24^), and its absence from the organoids indicates that these lack fully differentiated epithelial cells. Interestingly, superficial epithelial layers are also lost in the donor corneas in storage, and by RTPCR we did not detect *KRT*12 transcript in the donor cornea sample (Supplemental Figure 1 B). The *PAX6* homeobox gene, expressed in the RPE and neural cells is also present in the developing cornea^25^, and is expressed in C-ORGs (Supplemental Figure 1A). We also detected expression of markers for stromal keratocytes (*CD34*) and the ECM marker Keratocan (*KERA*), as well as putative endothelial markers (*COL8A1, F11R, S100A4*) (Supplemental Figure 1)^26^,^27^. The presence of these transcripts suggests that the organoid structures contain cells that *in vivo* would typically mark the three cell types that comprise the human cornea^3^,^28^. Additional immunofluorescence staining of selected markers further support partial differentiation of the organoids into multiple layers of the cornea as discussed below.

### Localization of corneal proteins by immunofluorescence

**Figure 4:**
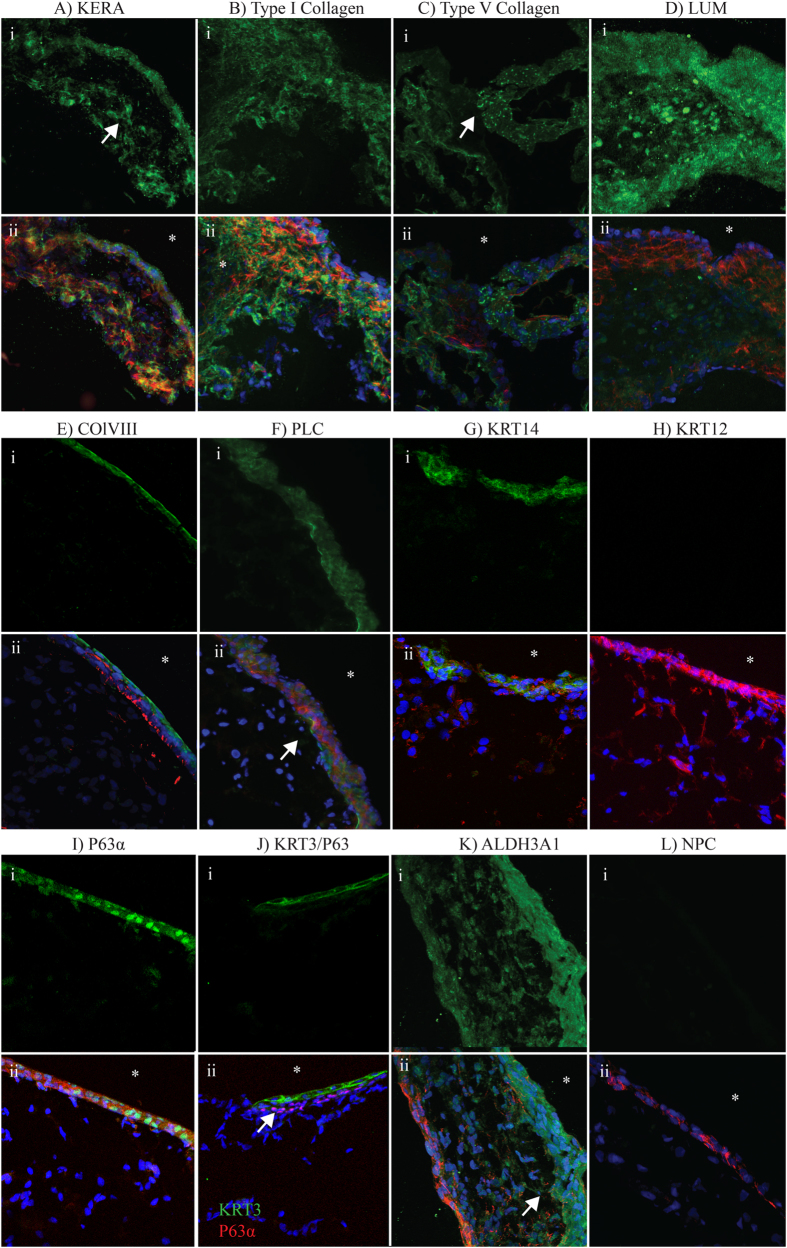
Immunofluorescence staining of ECM and crystallin proteins within corneal organoids. Staining for key corneal proteins in mature corneal organoids. Inlay (i) represents the protein of interest, inlay (ii) is overlayed with F-actin (Red) and nuclei (blue) except in inlay K. Regions of interest highlighted with arrows, “*” denotes external surface of the organoid. (**A**–**D**) Demonstrates presence of corneal stromal proteins. (**E**–**H**) Demonstrates markers of basement membrane and epithelial differentiation. (**I**) Shows expression of P63α in apical cells, (**J**) shows dual immunostaining for P63α in red whilst the differentiated epithelial marker KRT3 in greenin the apical cells above the P63 positive cells (arrow). (**K**) Indicated the corneal crystallin ALDH3A1 being expressed in both the most apical cells and the underlying “stromal” cells.

Cryo-sections of C-ORGs revealed a multi-layered architecture approximately 100 μm thick. The sections were immunostained for epithelial, stromal and endothelial markers (Fig. 4). The central multi-layered tissue segment is interspersed with cells immunopositive for stromal markers KERA, Collagen types I and V, and LUM (Fig. 4A–D). Collagen types I and V, are major fibrillar collagens^1^,^29^, and KERA^30^,^31^ and LUM^32^,^33^ are collagen associated keratan sulfate proteoglycans of the corneal stroma. To determine if the edges of the organoids carry epithelial basement membrane and endothelial Descemet’s membrane proteins we immunostained the organoids for collagen type VIII^34^ and perlecan^35^ (Fig. 4E,F). Collagen type VIII appeared more at the very outer edge while perlecan staining was somewhat diffused throughout the outer edge. KRT14 was detected in the apical cell layers of the organoids suggesting a primitive epithelial surface (Fig. 4G), this is further supported by the fact that we did not detect KRT12 in these superficial layers (Fig. 4H). Immunofluorescence staining for p63α showed positive staining of cells in the superficial layers of the cornea organoid while cells from the deeper layers were not stained (Fig. 4I). We also dually stained a section for p63α and KRT3 (Fig. 4J); while both appeared in the superficial layers, p63α appeared to be staining more of the basal cells. We also noted some KRT3 staining of the deeper layers in a second organoid (Supplemental Figure 2). The abundant corneal “crystallin”, ALDH3A1, is normally present in the epithelium and the stroma^36^. We found stronger staining for ALDH3A1 at one surface of the organoids, with weaker staining in the central layers (Fig. 4K). No primary control (NPC, L) is included to show antibody specificity.

### Transmission electron microscopy

**Figure 5:**
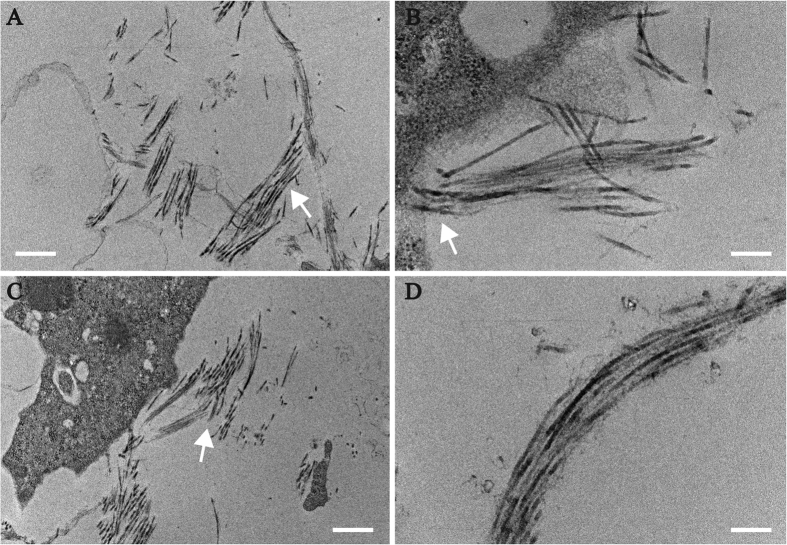
Collagen fibril accumulation in organoids. TEM showing arrangement of presumptive collagen fibrils viewed surrounding the cells. Collagen fibrils are highlighted with arrows in (**A**–**C**) a presumptive packed lamella with stacked collagen fibril is shown in (**D**). Scale bars A-0.5 μm, B-200 nm, C-0.5 μm, D-200 nm.

TEM of the organoids at low (Fig. 5A,C) and high magnifications (B, D) showed collagen fibrils within the ECM and some in the proximity of cells (B, C). The fibrils had small diameters with a homogeneous diameter distribution characteristic of stromal fibrils. The fibril assembled into small bundles (fibers) with rudimentary lamellar structure (D). The hierarchical assembly of fibrils, fibers and lamellae with an apparent angular displacement of adjacent structures is characteristic of stromal development^37^,^38^.

## Discussion

Results presented here support the development of a corneal organoid structure from human iPSCs. These organoids express markers of the three cell types of the cornea - epithelium, stromal keratocytes and the endothelium. Moreover, key extracellular matrix collagens and proteoglycan core proteins that make up the stromal matrix were present in the organoids. In the central organoid collagen fibrils were present. The fibrils demonstrated the beginning of a hierarchal structure with fibrils becoming organized into small bundles (fibers) often with a ~90 degree displacement of adjacent structures.

The emergence of the cornea organoids during the development of eye cups from iPSCs should be considered in the context of signals that regulate eye development. The vertebrate eye field develops under the regulation of the transcription factors TBX2/3/5^39^, RX, PAX6 and SIX3^5^. Retinoic acid signaling further participates in optic cup formation from optic vesicles. The culture conditions for developing optic cups from iPSC recreates an environment permissive for the development of the eye field and additional signaling that promotes neural fate. These early manipulations, supportive of an eye field, also allow development of surface ectoderm cells. It is conceivable that cells proximal to such ectodermal cells become neural crest-like to ultimately produce corneal endothelial- and keratocyte-like cells that produce collagen type VIII and stromal collagen types I and V, respectively.

As this study relies on cell mediated self-assembly and maturation of structures, the process of differentiation is orchestrated in a manner similar to that seen in development. In contrast, other tissue engineering approaches, such as culture of differentiated stromal fibroblasts in collagen matrix, hydrogels and other types of scaffolds attempt to recreate the local environment of the mature tissue. Alternatively, cell-based therapeutic approaches engage in transplanting limbal and mesenchymal stem cells and rely on their ability to populate and repair ocular surfaces. A recent study has published a 2-dimensional culture system whereby iPSCs demonstrated the ability to organize into a “self-formed ectodermal autonomous multi-zone” that could recreate different ocular epithelia^40^.

One previous study has reported the development of cell spheres termed “cornea orb” from a human embryonic stem cell line and from a second cell line derived from a parthenogenetic, unfertilized egg^41^. In that study the authors claim that the cornea orb had an epithelial- and an endothelial-side, characterized as being immuno-positive for mucin −1 and tight-junction protein ZO-1. Examined at the transcript level only, these corneal orbs were also positive for stromal genes (*LUM, KERA*) and *COL1A1* and *COLVA1*. Our study on the other hand used iPSCs to develop cornea organoids, where the corneal status was more stringently tested at the transcript and protein level for the presence of epithelial, stromal and endothelial markers. The organoids show remarkably appropriate immunofluorescence staining for epithelial markers KRT3, KRT14 and p63. The outer layers also show some extracellular staining of perlecan and collagen type VIII. Perlecan, a heparan sulfate proteoglycan is a major component of the epithelial basement membrane, but it is also present in the Descemet’s membrane of young corneas^42^. Collagen type VIII, on the other hand a major component of the Descemet’s membrane in the adult cornea is also expressed by epithelial cells of the cornea and the lens and various mesenchymal cells as well^34^. The deeper layers of the same cornea organoids also stain for stromal proteins LUM and KERA, Collagen types I and V. Most importantly, by TEM these organoids show organized collagen fibrils as seen in the corneal stroma.

The organoid field in general is moving towards using induced pluripotent stem cells rather than embryonic stem cells to develop various organoids such as intestinal, kidney, liver and retinal organoids^17^. Our findings indicate that conditions initially supportive of retinal organoid formation, at later stages can produce cornea organoids. This opens the cornea field to the development of 3D organoids for disease modeling, drug screens and ultimately gene editing and tissue replacement.

## Methods

### Cell Culture

All experiments were carried out in accordance with the relevant guidelines and regulations set out by the Johns Hopkins Stem Cell Research and Oversight committee. Normal donor anterior stromal caps in Optisol –GS (Bausch & Lomb, Rochester, NY) were obtained from endothelial keratoplasty from Tissue Banks International (Baltimore, MD) and the Indiana Lions Eye and Tissue Bank (Indianapolis, IN) under established guidelines related to informed consent for research use of human donor corneas. These experiments are covered by the Johns Hopkins Medicine IRB approved protocol entitled “Phenotypic and Genotypic Analysis of Keratoconus (NA_00006544).

Stromal cells from donor corneas were extracted by serial extraction with collagenase as described before^43^,^44^ and cultured in 1:1 DMEM:F12 with 10% fetal bovine serum, 1 mM phospho-ascorbic acid (Sigma-Aldrich, A8960), 1% antibiotic/mycotic (Gibco, 15240062) to maintain fibroblasts and in low glucose DMEM without serum and in the presence of insulin selenium and transferrin (ITS, Sigma, I3146), and 1 mM phospho-ascorbic acid, to maintain the keratocyte-like phenotype. The IMR90.4 iPSCs (WiCell) were grown under feeder-free condition in 5% O2 and 10% CO2 in mTeSR1 medium (StemCell technologies) on growth factor reduced Matrigel-GFR™ (#354230; BD Biosciences) as previously described (Wahlin, in review).

### Derivation of organoids

The IMR90.4 iPSCs maintained in mTeSR1 were passaged to a single cell density and 3,000 cells were added per well of a round bottom 96 well plate. After an overnight initial recovery period in mTeSR1 containing 5 μM blebbistatin in hypoxia (5% O_2,_ 10% CO_2_) cells were transitioned to a Neural induction medium: consisting of DMEM (#11965; Invitrogen) with 1% B27 vitamin A (-) (#12587010; Invitrogen) and 38.8 mg/L insulin (#11376497001; Roche), 128 mg/L L-ascorbic acid (#A8960; Sigma), 28 μg/l sodium selenium (#S5261; Sigma), 21.4 mg/L transferrin (#T0665; Sigma), 38.8 mg/L NaHCO3. Osmolarity was raised +30 mOsm to ~330–340 mOsm by adding 0.88 g/L NaCl. Aggregates were transferred at D10 to 15 ml tubes, rinsed 3× in HBSS, and treated with 100 nM Smoothened agonist (SAG; #566660; EMD Millipore) from D10–D18. Long term medium was a 3:1 mix of DMEM (Invitrogen #11965):F12 (#11765: Invitrogen) with 1% B27 (#17504044: Invitrogen), 10% heat inactivated FBS (#16140071; Invitrogen), 1 mM pyruvate (#11360; Invitrogen), 1xNEAA (#11140, Invitrogen), 1X Glutamax (#35050061; Invitrogen) and 1 mM taurine (#T-8691; Sigma). Vesicles were excised from D10–12 (as late as D16) with tungsten needles and fed every 2–3 days in suspension in ultra-low binding T-75 flasks (Corning) or untreated 10 cm polystyrene petri dishes. When 10 cm dishes were used, sedentary aggregates were monitored to ensure that they didn’t stick to the surface. Cells were maintained at 37 °C in standard 20% O2/5% CO2. To increase survival and differentiation, 500 nM all-trans retinoic acid (ATRA; #R2625; Sigma) was added to the medium from D20 until D120 (Fig. 1).

### RNA extraction and RTPCR

RNA was isolated from pooled organoids using the RNEasy kit (Qiagen, Valencia, CA) as per manufacturer’s instructions. 1 μg of RNA was reverse transcribed using the Superscript III RT kit (Life Technologies, Grand Island, NY). Quantitative PCR analysis was performed using the Bio-Rad CFX384 real time PCR machine, utilizing SYBR green chemistry (Applied Biosystems) 14 μl reaction volumes. Briefly, 20 nM of oligonucleotides were loaded with 3 ng of cDNA per reaction. The primer list and PCR conditions are provided in Supplemental Table 1.

### Immunofluorescence studies

The organoids were embedded in optimal cutting temperature compound (OCT) and flash frozen; then 10 μm sections were taken on a cryostat. These were allowed to air dry for 30 minutes before being fixed in 4% paraformaldehyde in PBS for 15 minutes at room temperature. The sections were treated with 0.1% Triton X-100 in PBS for 5 minutes at room temperature. The sections were then blocked in 3% BSA 2% normal goat serum for 1 hour. Primary antibodies were then added at appropriate concentrations overnight at 4 °C in a humidified chamber (Details provided in Supplementary Table 2). Slides were then washed 3 × 15 minutes in blocking buffer, secondary antibodies were diluted to 1:1000 in blocking buffer and then applied to the slides and allowed to incubate for 1 hour at room temperature. For visualization of the actin cytoskeleton, AlexaFluor-546 (Sigma) conjugated phalloidin sections were incubated at 1:200 in blocking buffer for 20 minutes at room temperature. Slides were then washed 3 times for 15 minutes and mounted in Vectashield containing DAPI (Vectorlabs, Burlingame, CA). Staining was visualized on a Zeiss Observer A1 fluorescent microscope (Zeiss GMBH, Jena Germany) or a Zeiss 510 M laser scanning confocal microscope. “No primary” controls were run to exclude non-specific staining.

### Transmission electron microscopy (TEM)

The organoids were analyzed for the presence of organized collagen fibril structures by TEM as described before^45^. Briefly the tissues were fixed in 4% paraformaldehyde, 2.5% gluteraldehyde, and 0.1 M sodium cacodylate pH7.4 in 8.0 mM CaCl2. After treatment with 1% osmium tetroxide, the tissues were dehydrated in serial dilutions of ethanol and then propylene oxide. The tissues were embedded in a mixture of Embed 812, nadic methylanhydride, dodecenyl succinic anhydride and DMP-30 (Electron Microscopy Sciences, Hatfield, PA). Sections (80 nm thick) cut with a Leica ultramicrotome, were stained with 2% aqueous uranyl acetate and 1% phosphotungstic acid, pH 3.2. The sections were viewed at 80 kV with a JEOL 1400 transmission electron microscope and a Gatan Orius widefield side mount CC Digital camera (Gatan Inc.).

## Additional Information

**How to cite this article**: Foster, J. W. *et al*. Cornea organoids from human induced pluripotent stem cells. *Sci. Rep.* **7**, 41286; doi: 10.1038/srep41286 (2017).

**Publisher's note:** Springer Nature remains neutral with regard to jurisdictional claims in published maps and institutional affiliations.

## Supplementary Material

Supplemental Data
