# Supplementary material for: Cornea organoids from human induced pluripotent stem cells
Source: Sci Rep. 2017 Jan 27;7:41286. doi: 10.1038/srep41286 (PMC5269590; doi:10.1038/srep41286)
Supplement: Supplemental Data [file srep41286-s1.pdf]

Supplementary Data

Title: Cornea organoids from human induced pluripotent stem cells

James W. Foster[1], Karl Wahlin[5], Sheila M. Adams[4], David E. Birk[4], Donald J. Zack[3], *Shukti Chakravarti[1,2,3]

Supplementary table 1.

Gene	*Accession number*	*Forward Primer*	*Reverse Primer*	*Product size*
CDH2	NM_001792.3	AAATTGAGCCTGAAGCCAAC	GTGGCCACTGTGCTTACTGA	102
CD34	NM_001025109	CTTGGGCATCACTGGCTATT	TCCACCGTTTTCCGTGTAAT	97
PAX6	NM_001310161	AAAGCAACAGATGGGCGCAGAC	TCTTTGCAGCTTCCGCTTCAGC	238
ABCG2	NM_001257386	TCCACTGCTGTGGCATTAAA	CCTGCTTGGAAGGCTCTATG	70
NP63	NM_003722.4	TGAGCCACAGTACACGAACC	TGCGCGTGGTCTGTGTTATA	99
KERA	NM_007035	TATTCCTGGAAGGCAAGGTG	ACCTGCCTCACACTTCTAGACC	102
KRT14	NM_000526.4	CGGCCTGCTGAGATCAAAGA	TCTGCAGAAGGACATTGGCA	106
COL8A1	NM_001850.4	AGATGCTGCTCTGCCTTCAC	AAGGGAGCTCACACGTTCAC	93
KRT12	NM_000223.3	CCATTGATGTCGGCCTCTAC	TTTCAGCCAGCATTGGAAA	130
F11R	NM_144502	GAAGAAAAGCCCGAGTAGGC	TCGAGAGGAAACTGTTGTGC	91
S100A4	NM_032034.3	ATGGAGGAGTTGGCAGTGTC	CTGCTTCCCTTGCAGAAAAC	148
POLR2A	NM_000937.4	CATCATCCGAGACAATGGTG	AACAATGTCCCCATCACACA	115
ΔNp63		GAA AAC AAT GCC CAG ACT CAA TTT	TCT GCG CGT GGT CTG TGT TAT	127
P63α		ATG TCG AAA TTG CTC AGG GAT TTT CAG A	TGA CCA CCA TCT ATC AGA TTG AGC ATT ACT	77

All quantitative PCR reactions were carried out under standard cycling conditions (40 cycles) with a 60°C annealing temperature.

P63 Primer sequences were obtained from Cavanagh et. al Experimental eye research, 2008, http://dx.doi.org/10.1016/j.exer.2007.12.007

Supplementary table 2

Antibodies used in figure 4

Target	*Raised in*	*Provider*	*catalogue number*	*Incubation concentration*
KERA	GT	Santa-Cruz biotechnology	SC-33244	1:200
COL1A1	RB	Novus	NB600-408	1:500
COLVA1	RB	Novus	NBP1-19633	1:500
COLLVIII	RB	Santa-Cruz biotechnology	sc-134947	1:500
ALDH3A1	RB	Abcam	ab76976	1:500
LUM	MS	Santa-Cruz biotechnology	sc-166871	1:200
PLC	RAT	Research Diagnostics	RDI-PERLECabrt	1:500
P63α	RB	Cell signalling	#13109	1:500
KRT12	RB	Abcam	AB185627	1:200
KRT14	RB	Abcam	Ab181595	1:200
KRT3	MS	Abcam	AB77869	1:200

Supplemental Figure 1

A. Raw gels used to generate figure 3. Bands represent amplified product after 40 cycles from qPCR experiments.

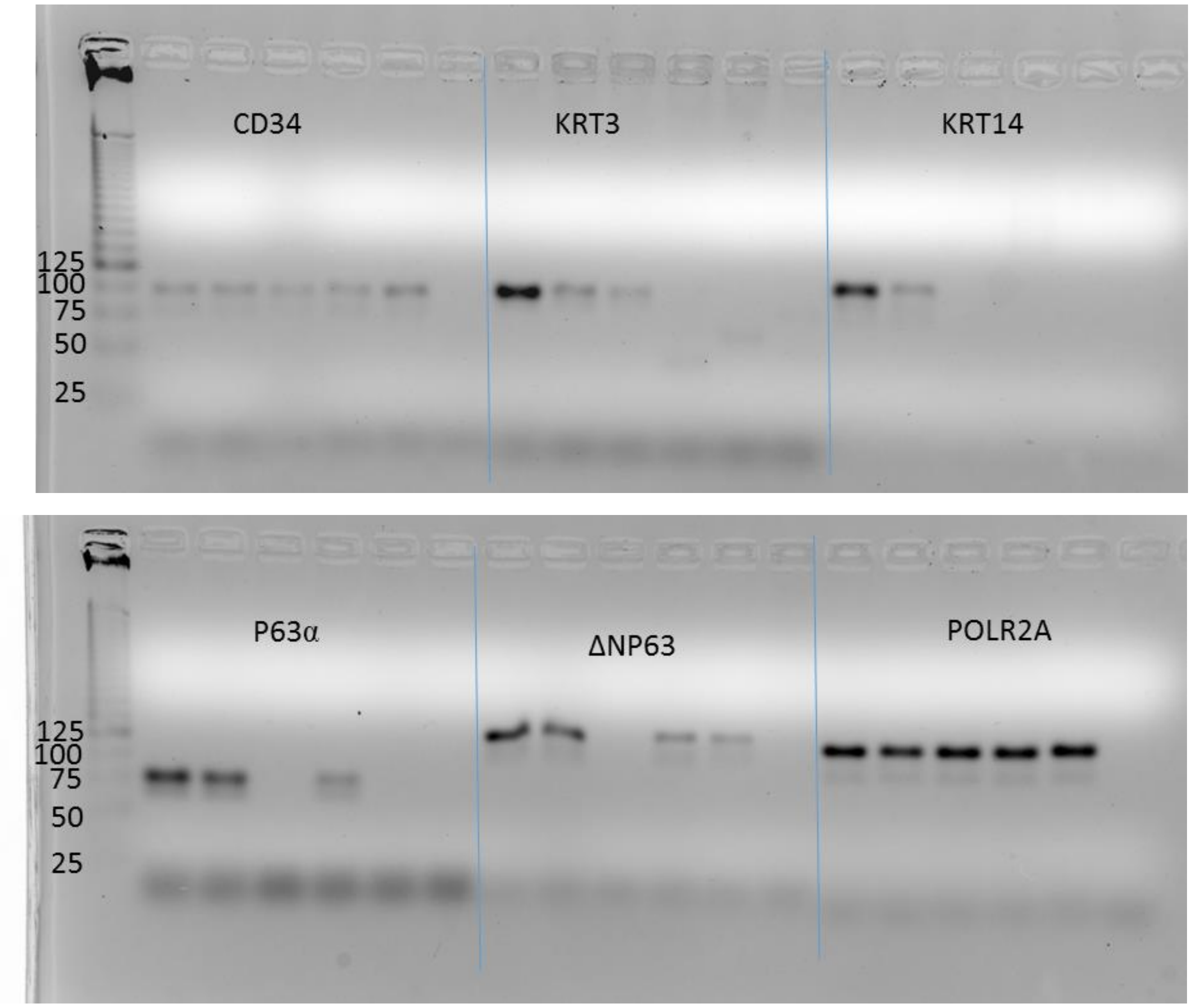

25bp ladder, (Thermo scientific, 10488-095)

Expected sizes:

CD34 – 99

KRT3 – 100

KRT14 – 100

P63α – 77

ΔNP63 – 127

B. Additional markers for corneal differentiation

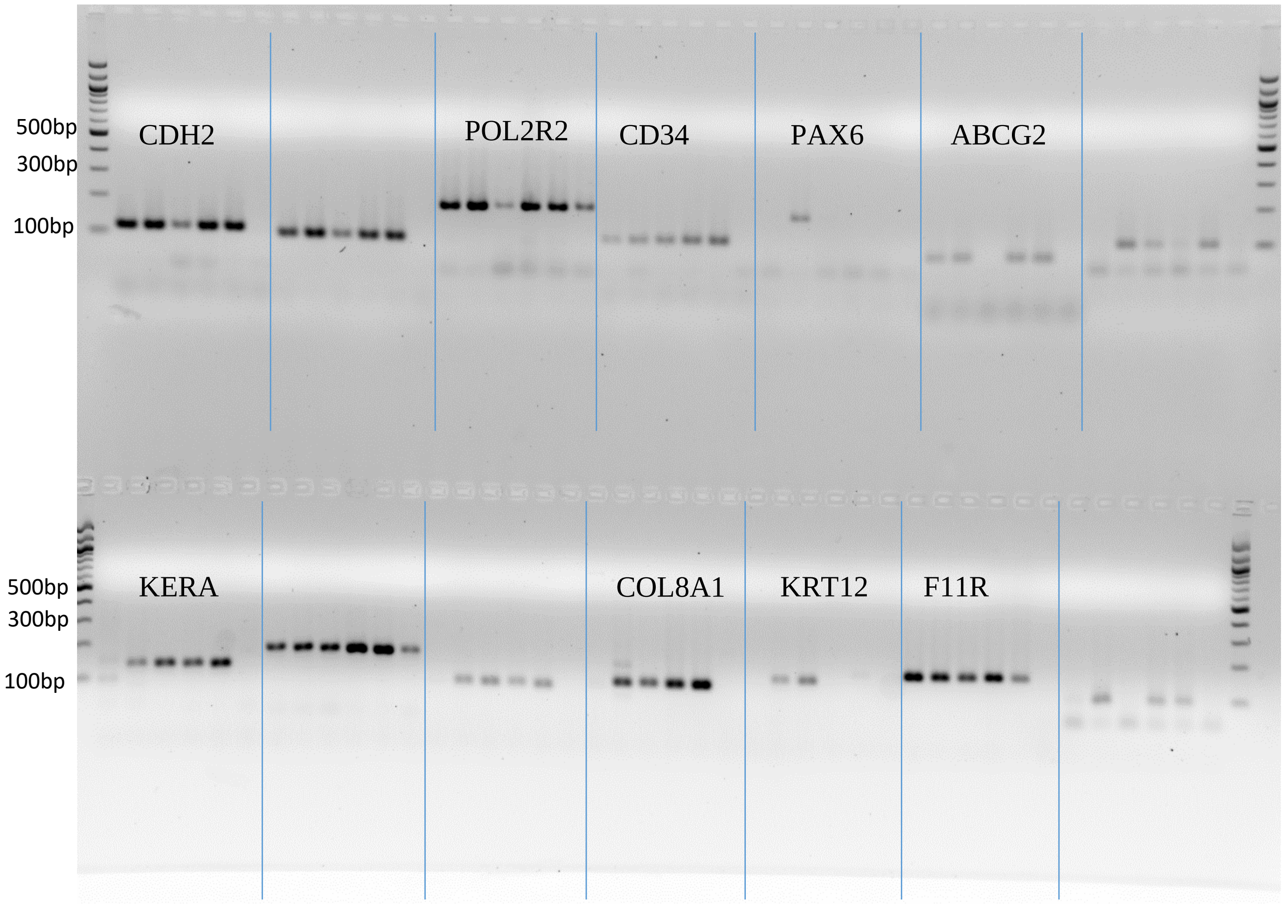

100bp ladder

Expected sizes.

CDH2	102
CD34	97
PAX6	238
ABCG2	70
KERA	102
COL8A1	93
KRT12	130
F11R	91
POLR2A	115

Unmarked lanes represent unexpected size products or positive RT control samples and have not been included, new sets of primers were used in some cases and are represented in Supplemental figure 1 A.

Supplemental Figure 2

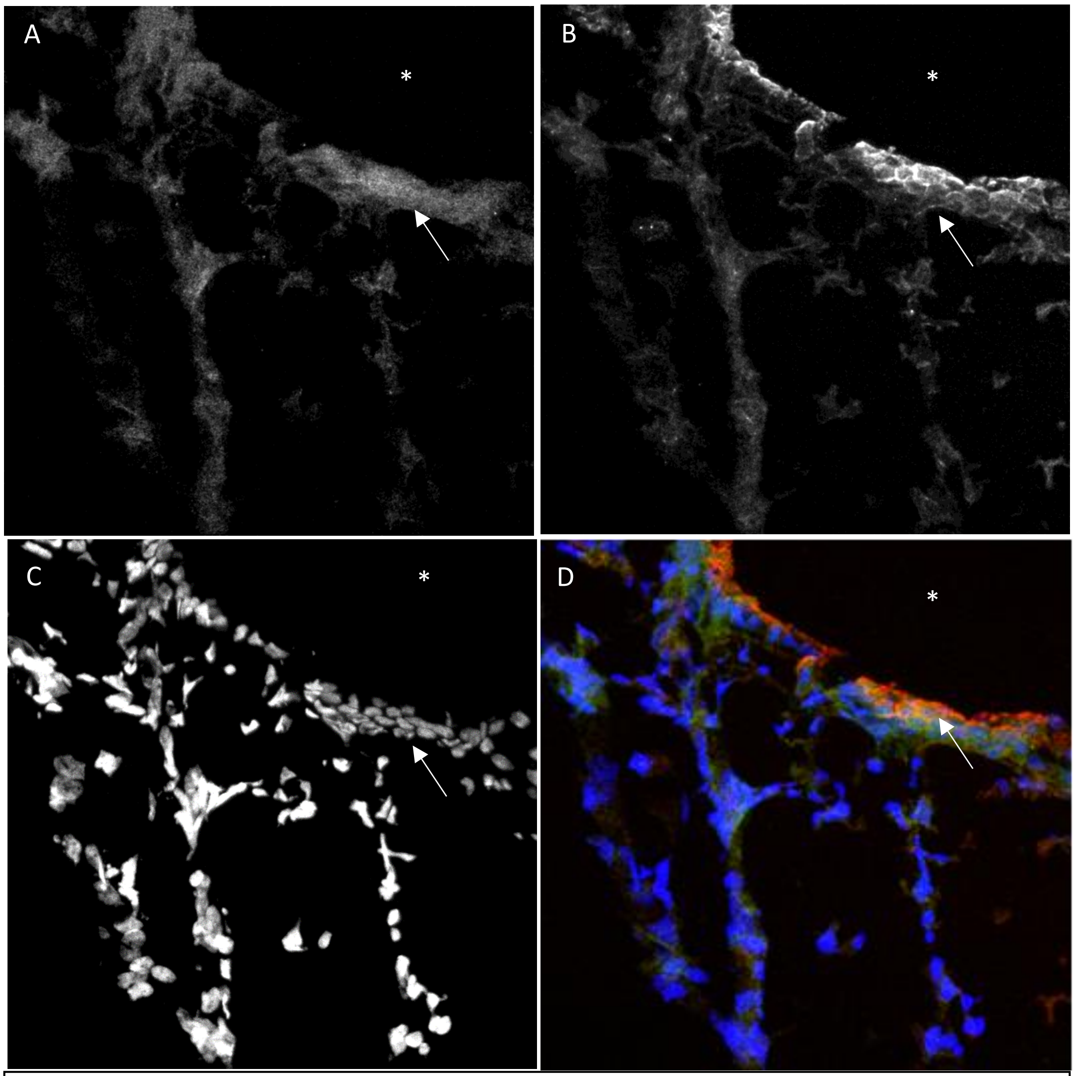

KRT3 staining of corneal organoid showing positive staining of basal layers. A – KRT3, B – F-Actin, C- DNA/Dapi, D – Merge: KRT3 (green), F-actin (Red), DAPI (blue). Note KRT3 staining in the dense, apical cell population, (Arrow).
